# Is care really shared? A systematic review of collaborative care (shared care) interventions for adult cancer patients with depression

**DOI:** 10.1186/s12913-019-3946-z

**Published:** 2019-02-14

**Authors:** Joanne Shaw, Suvena Sethi, Lisa Vaccaro, Lisa Beatty, Laura Kirsten, David Kissane, Brian Kelly, Geoff Mitchell, Kerry Sherman, Jane Turner

**Affiliations:** 1 The University of Sydney, Psycho-oncology Co-operative Research Group, School of Psychology, Faculty of Science, Sydney, NSW 2006 Australia; 2 College of Medicine & Public Health, Flinders Centre for Innovation in Cancer, Adelaide, SA Australia; 3 Nepean Cancer Care Centre, Sydney, NSW Australia; 4 Department of Psychiatry, Monash University, Melbourne, Vic Australia; 5 School of Medicine & Public Health, The University of Newcastle (UoN), Callaghan, NSW Australia; 6 Faculty of Medicine, The University of Queensland, Herston, Qld Australia; 7 Centre for Emotional Health, Department of Psychology, Macquarie University, Sydney, NSW Australia; 8 Discipline of Psychiatry, School of Medicine, The University of Queensland, Herston, Qld Australia

**Keywords:** Collaborative care, Shared care, Systematic review, Depression, Cancer, Randomised controlled trial

## Abstract

**Background:**

Collaborative care involves active engagement of primary care and hospital physicians in shared care of patients beyond usual discharge summaries. This enhances community-based care and reduces dependence on specialists and hospitals. The model, successfully implemented in chronic care management, may have utility for treatment of depression in cancer. The aim of this systematic review was to identify components, delivery and roles and responsibilities within collaborative interventions for depression in the context of cancer.

**Methods:**

Medline, PsycINFO, CINAHL, Embase, Cochrane Library and Central Register for Controlled Trials databases were searched to identify studies of randomised controlled trials comparing a treatment intervention that met the definition of collaborative model of depression care with usual care or other control condition. Studies of adult cancer patients with major depression or a non-bipolar depressive disorder published in English between 2005 and January 2018 were included. Cochrane checklist for risk of bias was completed (Study Prospero registration: CRD42018086515).

**Results:**

Of 8 studies identified, none adhered to the definition of ‘collaborative care’. Interventions delivered were multi-disciplinary, with care co-ordinated by nurses (*n* = 5) or social workers (*n* = 2) under the *direction* of psychiatrists (*n* = 7). Care was primarily delivered in cancer centres (*n* = 5). Care co-ordinators advised primary care physicians (GPs) of medication changes (*n* = 3) but few studies (*n* = 2) actively involved GPs in medication prescribing and management.

**Conclusions:**

This review highlighted joint participation of GPs and specialist care physicians in collaborative care depression management is promoted but not achieved in cancer care. Current models reflect hospital-based multi-disciplinary models of care.

**Protocol registration:**

The protocol for this systematic review has been registered with PROSPERO. The registration number is CRD42018086515.

**Electronic supplementary material:**

The online version of this article (10.1186/s12913-019-3946-z) contains supplementary material, which is available to authorized users.

## Background

A diagnosis of cancer impacts individuals’ psychological and physical wellbeing. Prevalence estimates of major depression (16%), minor depression and dysthymia (22%) in cancer patients are higher than in the general population. [1, 2] In palliative settings, the prevalence of depression approaches 49%. [3] Inadequate treatment of depression results in poorer adherence to anti-cancer treatments, decreased tolerance of cancer treatment side-effects, higher use of health care resources including increased hospital re-admissions, may adversely impact on interpersonal relationships and reduced overall survival. [3–9] Co-existing depression therefore poses a significant burden for patients, families and the health system. Cognitive behavioural therapy (CBT) is effective in treating depression [10] with a meta-analysis (*n* = 198 studies, 22, 238 patients) reporting medium to large effect sizes sustained 6–12 months post intervention. A recent meta-analysis also confirmed the effectiveness of antidepressant medication in the treatment of depression [11] both in combination with psychological therapy and as primary treatment. [12] To guide evidence-based treatment, there has been a renewed effort to promote routine distress screening as a first step to improve detection and hence treatment for depression in cancer care. [13] Such strategies have included the development of a clinical pathway for identification and management of depression in adults with cancer, an international first. [14]

Despite increased evidence about effective treatments, many patients still do not seek treatment for their depression. For instance, a meta-analysis of 53 studies (*n* = 12,052) found in a research context less than 60% of distressed cancer patients engage in psychological treatment. [15] Uptake in routine care is even lower. [16] At a system level access to treatment is constrained by a shortfall in the psycho-oncology workforce resulting in long waiting lists and geographical disparities in access. [17–19] Practical constraints such as transport, inconvenience and cost have also been suggested. [20, 21] Normalisation of distress and attribution of somatic depression symptoms to cancer by clinicians also means depression goes untreated and patients’ attitudes to mental illness are also likely to play a role. [22] Patient factors such as negative attitudes to mental health and stigma also reduce patient willingness to access treatment. [23]. To address perceived barriers, models of care that encompass systematic identification of depression in cancer patients, reduce dependence on specialist input and enable timely access to evidence-based treatment have been proposed.

The collaborative care model, based on the principles of chronic disease management, has been successfully implemented to treat medical conditions including depression. [24, 25] Core components of collaborative care for depression are: i) a multi-professional approach to patient care, ii) a structured management plan tailored to depression symptom severity, iii) scheduled patient follow-ups and iv) enhanced inter-professional communication. [26] The model endorses a multi-disciplinary approach involving joint participation of primary and specialist care physicians in planned delivery of care over and above routine discharge and referral. [23] A key aspect of effective collaborative care is case management [27] in which a member of the clinical team works closely with the main treating physician and monitors patient progress including adherence to psychological and pharmacological treatments, initiating treatment changes as necessary. [28] A meta-analysis of collaborative care interventions (*n* = 37 studies, 12,355 patients with depression receiving primary care) found that interventions that included a mental health professional as a care provider within the clinical team reported the largest effect sizes. [29]

The evidence base for collaborative care for depression among patients with cancer is rapidly developing. A recent meta-analysis of studies (*n* = 8) purporting to be collaborative care interventions concluded that the interventions were significantly more effective than usual care (standardized mean difference = − 0.49, *p* = 0.003), with remission rates for depression higher in the intervention groups at 12 months. While promising, the collaborative care interventions identified in that meta-analysis varied in content, intensity, and number of components, thus making it difficult to determine the relative contribution of each component to overall effectiveness. [30] Prior to wider implementation of the model, greater understanding of the determinants essential to model success for the treatment of depression in oncology is required. The aim of this systematic review was to determine the fidelity of the depression collaborative care models trialled in oncology to the recommended collaborative care criteria. Specifically, the review sought to identify reported intervention components, delivery models and role responsibilities.

## Methods

### Study eligibility

#### Types of studies

Studies presenting primary data from randomised controlled trials (RCTs) of adult cancer patients with major depression or a non-bipolar depressive disorder, which compared a treatment intervention designated as a collaborative (or shared) model of depression care with usual care or other control group and published in English were included. Non-randomised, single-arm, case control studies, qualitative studies and case-series reports were excluded.

#### Participants

Studies were eligible if participants were over 18 years of age with a current or prior diagnosis of cancer (excluding basal cell carcinoma and squamous cell carcinoma) and where the study population met a threshold for clinical depression on a validated depression measure or structured clinical interview. Studies including separate sub-group analyses of patients that met the criteria for clinical depression were also eligible for inclusion.

#### Interventions

Collaborative care, defined as a treatment approach integrating primary- and tertiary-level (hospital) care in the management of co-morbid depression in cancer patients.

#### Controls

Treatment as usual, wait-list control groups, and other treatment interventions.

#### Outcomes

Studies reporting data on the efficacy of collaborative care interventions.

### Search strategy

Medline, PsycINFO, CINAHL, Embase and the Cochrane Library and Central Register for Controlled Trials databases were searched using the keywords and MeSH terms [Depression/depressive disorder/ or depressive disorder, major/or dysthymic disorder/] and [Cancer or Carcinoma or Neoplasm] and [Collaborative Care or Shared Care or Integrative Care] and randomised controlled trials for English language articles published between January 2005 to January 2018 as the first collaborative care intervention for depression in cancer was published in 2005. Primary studies presenting data from RCTs of adult cancer patients with major depression or a non-bipolar depressive disorder that compared care delivered in a collaborative care model were identified. See Additional file 1: Table S1 for an example database search strategy. Reference lists of retrieved articles and previous systematic reviews were also searched for relevant publications. Searches were conducted for outcome data for published study protocols identified.

### Data abstraction

**Fig. 1:**
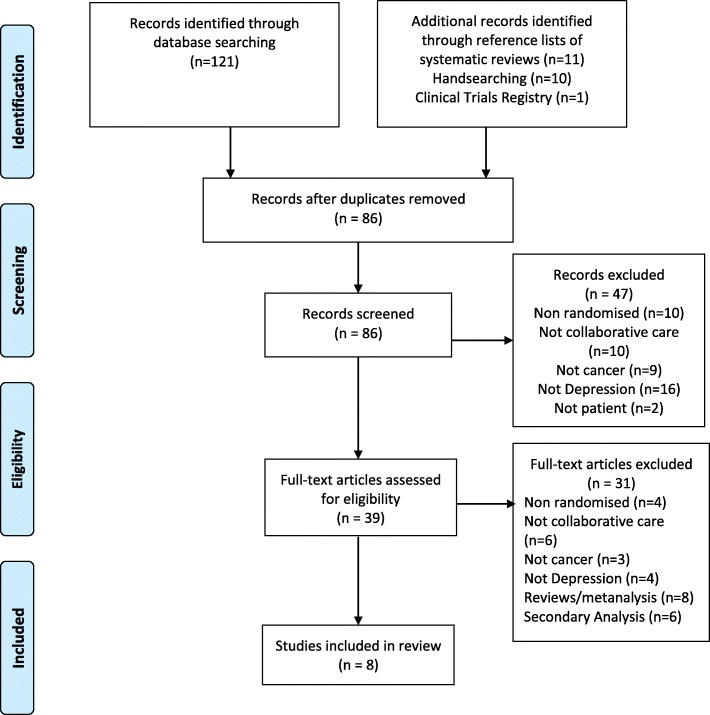
(PRISMA diagram): Search process for the review (as at January 2018)

Abstracts were identified and independently reviewed by two reviewers (SS and LV). Data extraction was independently conducted by three reviewers (SS, LV and JS) and coding disagreements arising were discussed and consensus coding applied. A coding framework was developed to extract the components of collaborative care based on the key criteria defining collaborative care models: i) multi-professional patient care, ii) a structured stepped care management plan, iii) scheduled patient follow-ups and iv) enhanced inter-professional communication [23] Specifically, the following information was extracted for each study: study characteristics, including study aim(s), population, design and primary outcome(s); intervention description, follow up protocols and role of health professionals. Reasons for study exclusion are listed in Additional file 2: Table S2. The review methodology undertaken adhered to PRISMA guidelines for systematic reviews [31] and the search process is summarised in Fig. 1. Risk of bias was determined based on the Cochrane criteria (Additional file 3: Table S3). [32]

## Results

### Study characteristics

Eight primary studies met the review inclusion criteria. Mean sample size was 281 patients (SD 161.04; range 55–500). Cancer populations included lung (*n* = 1) [33], upper gastrointestinal/liver (*n* = 1) [34], mixed breast and gynaecological (*n* = 2) [34, 35] or heterogeneous (*n* = 4) [36–39]. Participants had major depressive disorder (MDD) (*n* = 3) [33, 36, 40], MDD or dysthymia (n = 2) [37, 38], MDD, dysthymia or persistent depressive symptoms > 1 month (*n* = 1) [35] and depressive symptoms based on self-report questionnaires only (*n* = 2) [34, 39] Greater than 60% female participation was reported in six studies [33, 35–37, 39, 40] primarily due to an over-representation of breast cancer patients. Of the included studies three were conducted by a single group in Scotland [31, 34, 35] and the remainder were conducted by separate groups in the USA. Study Characteristics are listed in Table 1.

**Table 1:** Characteristics of Included Studies

Author, Year Country	Study Design and comparator group(s)	Study Aims	Primary outcome	Sample Size and % females	Mean Age (SD)	Cancer Type	Depression	Setting	Collaborative care protocol
Dwight-Johnson [35] (2005) USA	Randomised pilot study comparing collaborative care intervention with usual care	Determine whether primary care collaborative depression be adapted and implemented in public sector oncology clinics serving low-income Latino patients	≥50% improvement in depression score (PHQ9) at 8 months	55 (100% female)	47.2 (11.3)	Breast or cervical, mixed stage	MDD dysthymia or had persistent depressive symptoms at baseline & 1 month	Cancer centre	IMPACT
Strong [36] (2008), Scotland	Single centre proof of concept RCT comparing collaborative care intervention with usual care	Investigate whether usual care plus depression care for people with cancer (intervention) could achieve a greater reduction in depressive symptoms at 3 months compared to usual care alone, and whether this would be sustained at 6 and 12 months. (SMART1)	self-reported depressive symptoms (SCL-20D) at 3 months	200 (71% female)	56.6 (11.8)	Heterogeneous stage not reported	MDD	Cancer care centre	Depression Care for People with Cancer
Ell [37] (2008; 2011) USA	Single centre efficacy RCT comparing collaborative care intervention with Enhanced usual care (standard oncology care plus psycho-educational pamphlets; list of center/community financial, social services, transportation, and childcare resources)	Determine the effectiveness of the Alleviating Depression Among Patients With Cancer (ADAPt-C) collaborative care management for major depression or dysthymia	Treatment response at 12 months; defined as 50% or a 5-point reduction of PHQ-9 score	472 (85% female)	TBC	Heterogeneous stage mixed	MDD or dysthymia	Cancer centre	IMPACT
Fann [38] (2009 USA)	Multicentre RCT comparing collaborative care intervention with usual care	Examine the effectiveness of collaborative care (IMPACT) for depression in in older primary care patients	treatment response defined ≥50% reduction in SCL-20 score at 12 months	215 (60% female)	71.75 (0.5)	Mixed type and stage	MDD or dysthymia	Primary Care	IMPACT
Kroenke [39] (2010) USA	RCT comparing collaborative care intervention with usual care	To determine whether centralized telephone-based care management coupled with automated symptom monitoring can improve depression and pain in patients with cancer.	Reduction of ≥50% in depression severity; (HSCL-20) at 12 months.	405 (309 depression; 68% female)	58.8 (10.8)	Mixed type and stage	depressed mood, anhedonia; or both. Moderately severe based on PHQ9	Telephone	Study specific
Sharpe [40] (2014) Scotland	multicentre effectiveness and cost effectiveness RCT comparing collaborative care intervention with usual care	Establish whether depression care for people with cancer (intervention) is better than usual care in achieving a clinically useful improvement in depression (SMART 2)	‘treatment response’ measured at 24 weeks; defined as a reduction of ≥50% baseline depression score, measured via Symptom Checklist (SCL-20D)	500 (90% female)	56.3 (10.1)	Breast, gynaecological, genitourinary; ‘good prognosis’	MDD	Cancer care centre or primary care clinic	Depression Care for People with Cancer
Walker [33] (2014) Scotland	Multicentre efficacy RCT comparing collaborative care intervention with usual care	Assess the efficacy of an integrated treatment programme (depression care for people with cancer) for major depression in patients with lung cancer compared with usual care. (SMART 3)	average depression severity during trial participation: participant’s depression severity averaged over the time from randomisation up to a maximum of 32 weeks	142 (65% female)	63.7 (8.8)	Lung; ‘poor prognosis’	MDD	Patient’s home or cancer centre/hospice	Depression Care for People with Cancer
Steel [34] (2016) USA	Multicentre efficacy RCT comparing collaborative care intervention Enhanced usual care (usual care + if a patient scored high on the CES-D care coordinator provided education about the symptoms and referrals to a mental health professional/ GP)	Examine the efficacy of a collaborative care intervention in reducing depression, pain, and fatigue and improve quality of life	Reduction in depression (CES-D) at 6 months	261 (82 depression; 27% female)	61 (11)	Upper GI cancer or other primary cancers with liver mets	No specific eligibility criteria for depression; subgroup analysis of patients with CES-D > 16 at baseline	Telephone and oncology outpatient clinic	Study specific

### Collaborative care interventions

#### Study setting

In contrast to depression collaborative care models more broadly, five studies delivered the intervention within the cancer centre, [34–37, 39] with a single study conducted in primary care. [38] A further study allowed patients to choose either the cancer centre or primary care for treatment[36]and another study in advanced cancer provided an option for care to be delivered in the patient’s home. [40] For two studies conducted in cancer clinics much of the care was provided via telephone/ web-based as well as face to face blended models. [34, 39] See Table 1 for study setting.

#### Model components

Classification of studies based on collaborative care interventions found three studies [33, 40] (collectively called the SMART studies) utilised variations of a Depression Care for People with Cancer (DCPC) intervention. [41] Two studies [35, 38] implemented the Improving Mood – Promoting Access to Collaborative Treatment program (IMPACT), a stepped care management program developed for treatment of depression in older primary care patients and a third study (ADAPT-C [42]) adapted the IMPACT protocol to incorporate a greater patient navigation role for Latino cancer patients. [37] A further two studies reported study-specific blended face to face and telephone [39] or web-based [34] collaborative care interventions. The key components of each intervention are listed in Table 2.

**Table 2:** Health Professional Roles in Collaborative Care Interventions

Study	Treatment Initiation/Psychoeducation	Treatment Planning	Psychological Treatment	Antidepressant Management (AM)	Treatment phase	Maintenance phase	Treatment Review	Maintenance Review	Follow Up
Dwight-Johnson [35] (2005)	Education session delivered by Cancer/Depression Clinical Specialist (social worker) included: importance of depression treatment to cancer treatment adherence, overall health and wellbeing; education about anti-depressants and problem-solving therapy	Patients choose either anti-depressant or problem-solving therapy as first line therapy. Treatment plan was recorded in the medical record	Problem-solving intervention weekly sessions with the social worker	initial meeting with patient, oncologist and SW to initiate treatment. Psychiatrist was available for same day consultation as required. Oncologist provided medication follow up during regularly scheduled clinic visits	8 weeks/sessions	8 months	2 weeks	Regular oncology clinic visits	SW follow up every 2 weeks – side effects, medication adherence, depression symptoms for 8 weeks SW provides feedback to oncologist and psychiatrist Patients who did not experience 50% reduction in symptoms after 8 weeks were scheduled for a consultation with psychiatrist to make treatment adjustments Results of consultation were feedback to oncologist and SW. Medication follow up conducted by psychiatrist or oncologist
Fann [38] (2009)	Depression Care Manager (DCM - a nurse or clinical psychologist) conducted a psychosocial history, provided education and behavioural activation	Patients offered depression management by a depression care manager working collaboratively with the patient and primary care physician in the patient’s usual primary care clinic Patients identify treatment preferences	Structured six- to eight-session psychotherapy program: Problem-Solving Treatment (PST), behavioural activation in Primary Care delivered by the DCM	Prescribed by the patients’ primary care clinician based on a stepped-care pharmacotherapy algorithm recommending routinely available antidepressant medications	Upto 10 (45 min) structured sessions over 3 months	3 months	In line with session schedule	Monthly PHQ9	In person or telephone follow-up every 2 weeks during acute-phase treatment, with subsequent monthly contact during continuation and maintenance phases
Ell [37] (2008)	The initial cancer depression clinical specialists (CDCS; bilingual social workers) conduct a semi structured psychiatric/psychosocial assessment; patient depression, psychotherapy, and antidepressant education; consideration of initial treatment choice; and provision of patient navigation assistance and included family members at patient request.	A personalized treatment plan that included patient AM or problem-solving therapy (PST) preferences. After acute treatment, patients received a treatment maintenance and relapse prevention program, including CDCS monthly telephone contacts up to 12 months after treatment	CDCS provided Problem Solving Therapy; Weekly sessions ranging from 6 to 12 weeks. Community services navigation was also provided	Psychiatrist prescribed	6–12 weeks	12 months	Not specified	monthly	CDCS telephone maintenance/relapse prevention and outcomes monitoring over 12 months.
Strong [36] (2008)	Patients screened in outpatient cancer clinics to identify MDD	Patient’s primary-care doctor and oncologist informed of the diagnosis and provided with advice on choice of antidepressant drug if requested.	Nurse-delivered a maximum of 10 one-to-one sessions over 3 months. The content of the intervention comprised education about depression and its treatment, problem-solving treatment (PST)	Primary-care doctors prescribed AMs. If the patient decided, during discussions with the nurse, to start or change AM, they were encouraged to contact their primary care doctor for this purpose. GP contacted by the nurse (by fax or telephone) to provide information about the patient and advice from a study psychiatrist.	Stepped care model: Step 1–10-12 weeks Step 2: a further 10 weeks Step 3: psychiatric referral	12 months	Weekly or bi-weekly	monthly	For 3 months after the treatment sessions progress was monitored by monthly telephone calls
Sharpe [40] (2014)	Nurses establish a therapeutic relationship with the patients, provide information about depression and its treatment,	Psychiatrists supervise treatment. They advise primary care physicians about AM prescribing and provide direct consultations to patients who are not improving.	Nurse delivered brief evidence-based psychological interventions (problem-solving therapy and behavioural activation) and monitor patients progress	If the patient chooses to try medication, the care manager liaises with their GP regarding a prescription. The supervising psychiatrist may make a recommendation to the GP regarding the choice of medication, based on the profile of the patients’ depressive symptoms, potential side effects and possible interactions with other drugs.	3 telephone calls over 12 weeks	12 months	Automated monitoring: twice a week for the first 3 weeks, then weekly during weeks 4 through 11	twice a month during months 3 through 6, and once a month during months 7 through 12	
Walker [33] (2014)	The depression care for people with lung cancer treatment programme is adapted to include strategies to achieve a rapid treatment response and to enable the patient to continue treatment despite physical deterioration. Nurses establish a therapeutic relationship with the patients, provide information about depression and its treatment and monitor patients’ progress	Psychiatrists supervise treatment, and provide direct consultations to patients who are not progressing	Nurses deliver brief evidence-based psychological interventions (problem-solving therapy and behavioural activation) in 10 structured sessions over 4 months in the persons home.	Psychiatrists supervise treatment, advise primary care physicians about prescribing to ensure rapid initiation and proactive adjustment of antidepressants, and provide direct consultations to patients who are not progressing	Upto 10 sessions over 4 months	8 months	In line with session schedule	Monthly Automated PHQ9	The nurse monitors the patient’s PHQ-9 scores monthly by telephone for a further 4 months and provides additional sessions for patients who do not meet treatment targets
Kroenke [39] (2010)	Participants undergo automated symptom monitoring by either telephone or the Internet, depending on their preferences. Participants can receive scheduled (automated) calls from the system (outbound), can initiate calls themselves to the system if these are more convenient (inbound) or, if they have a personal computer, can enter a secure Web site to complete their surveys. All participants receive an initial call (Week 0) to assess symptom severity and initiate treatment and a follow-up call in 1–2 weeks to assess symptom severity, adherence and adverse effects. Participants with depression receive two additional DPCM follow-up calls in the first 12 weeks of treatment	the nurse care manager recommends treatment for symptoms in accordance with evidence-based guidelines and monitors response and adherence	Telephonic care management was delivered by a nurse care manager trained in assessing symptom response and medication adherence; in providing pain and depression specific education; and in making treatment adjustments according to evidence-based guidelines	The oncologist implements treatment recommendations based on antidepressant algorithms. Treatment recommendations were provided to the study participant’s oncologist who was responsible for prescribing all medications and the psychiatrist becomes directly involved in the management of difficult cases	Upto 10 30-45 min sessions in 16 weeks	4 months	In line with session schedule	Monthly PHQ9	Participants received a baseline and 3 follow-up calls (1, 4, and 12 weeks) during the first 3 months of treatment. In addition to these scheduled telephone contacts, triggered telephone calls occurred when automated monitoring indicated inadequate symptom improvement, nonadherence to medication
Steel [34] (2016)	The medical team referred each patient and a psychiatric intake conducted by the care coordinator (psychologist) The web-based collaborative care intervention included access to a psychoeducational web site and to a collaborative care coordinator. The website included (1) psycho-educational information with regard to depression, pain, fatigue, nausea and vomiting and sleep; (2) a self-management area where the patient could record their symptoms and monitor changes through graphical depictions; (3) an area for journaling; (4) a chat room that connected the patient to other patients enrolled in the study, (5) an audiovisual library that included relaxation techniques and educational videos by the patient’s nurse coordinators; and (6) resource library.	The patient had telephone contact with the care coordinator approximately every 2 weeks and face-to-face contact with the care coordinator in the oncology outpatient clinic and/or hospital approximately every 2 months.	The care coordinator provides CBT and/or recommendations for pharmacological management of symptoms if the patient preferred medication to CBT or in addition to CBT.	The care coordinators provided information to the medical team about any changes in a patient’s symptoms that might have warranted changes in treatment. The care coordinator would discuss with the patient if s/he was interested in changing their treatment. The medical team may or may not have accepted the care coordinators’ recommendations	2 weekly telephone and monthly face to face	6 months	Not stated	Not stated	The care coordinators would have face-to-face contact with each patient in the outpatient cancer clinic or in the hospital when the patient visited the hospital for follow-up or treatment. The care coordinators contacted patients by phone but were also available as needed to the patients for questions and concerns.

All interventions provided psychoeducation and the majority (*n* = 7) included psychological therapy and/or anti-depressant medication. For the studies based on the IMPACT intervention, patient preference influenced delivery of first line psychological *or* anti-depressant therapy. [35, 37, 38] The SMART studies [33, 36, 40] recommended *both* psychological therapy and anti-depressant medication, if indicated. Similarly, treatment associated with the web-based collaborative care model was based on patient preference for cognitive behavioural therapy (CBT), antidepressant medication or both [34] and medication alone was considered first line treatment in the Kroenke trial. [39] In those studies where psychological treatment was a component of the intervention, treatment length ranged from 8 weekly sessions [33] up to 10 sessions over 3–4 months [31, 34, 35] with Problem Solving Treatment (PST) and Behavioural Activation the most common psychological interventions. [33, 35–38, 40] A single study characterised their therapy as CBT. [34] One study did not include a psychological therapy option as part of the treatment plan. [39] Six studies reported data on psychological treatments received. [33, 35, 36, 39, 40] Rate of uptake for psychological treatment in the intervention groups were higher than control groups and ranged from 5 to 98%. [33, 35–37, 40] Studies reliant on patient preferences for *either* psychological or pharmacological depression treatment reported lower rates of uptake (5–43%). [35, 37] Similarly, rates of antidepressant use were higher than controls and ranged from 35 to 85% of participants at 6 months. [33, 36, 37, 39, 40]

Five studies included training in intervention delivery for care co-ordinators [33, 34, 36, 37, 40] and a single study documented training for oncologists responsible for antidepressant management. [35] Care co-ordinators’ fidelity to the intervention manual was formally assessed in four studies [33, 36, 37, 40] although fidelity assessment and ongoing education were not included as part of the protocol for studies where the role of prescribing was delegated to oncologists and GPs.

#### Inter-professional roles within the collaborative care model

With respect to multi-professional involvement, all studies included a care co-ordinator and a mental health specialist as the primary members of the care team. Across studies, delivery of the psychological/psychoeducation components of the intervention as well as patient liaison and assessment of treatment adherence was undertaken by an appropriately trained nurse (*n* = 4), [33, 36, 39, 40] social worker (*n* = 2) [35, 37] or a psychologist (*n* = 1) [34] with one study including both a nurse and a psychologist. [38] The care co-ordinators all underwent formal study-specific training and had weekly [33, 34, 36–40] or bi-weekly [35] supervision to review patient progress and initiate treatment adjustments, as required. Supervision was generally provided by a psychiatrist (*n* = 7), although in one study a clinical psychologist was the appointed supervisor. [32]

Despite being classified as collaborative care interventions, studies deviated from the model in terms of wider inter-professional involvement in treatment, with four studies reporting oncologists were typically *informed* of the patient’s diagnosis but had minimal engagement with respect to treatment decision-making. [33, 36, 37, 40] In one study [35] the psychiatrist provided advice to the oncologist with respect to anti-depressant treatment adjustment/ follow up and in a second study, the oncologist followed evidence-based anti-depressant prescribing and management algorithms, with little input from the psychiatrist. [39] Similarly, three studies reported little or no primary care (GP) involvement in treatment decision-making and no documented contact with the GP by the treating team. [35, 37, 39] In the studies utilising the DPCP intervention (SMART studies), [31, 34, 35] although the GPs were responsible for anti-depressant prescribing, clinical decisions and recommendations for medication treatment and/or adjustment were made by the psychiatrist, with little engagement with the GP around treatment planning. A single study reported GP prescribing based on evidence-based algorithms [38] and one study divided prescribing responsibility between the medical team and the patient’s GP, although the role of each clinician was not clearly defined. [34] Table 3 lists the health professional roles across studies.

**Table 3:** Collaborative Care Intervention Components

Authors (year)	Care Co-ordinator	General Practitioner	Oncologist	Psychiatrist	Interdisciplinary Communication	Intervention Training	Fidelity Assessment
Dwight-Johnson [35] (2005)	Social workers (CDCS) carry out the majority of treatment: problem solving treatment (PST); patient navigation/case management; monitoring, follow up.	No role	antidepressant prescribing	Advice to oncologist; medication follow up; bi-weekly supervision to CDCS.	CDCS provides feedback to oncologist and psychiatrist.	Oncologist provided with two 1-h education sessions by psychiatrist on depression. Given summarised pocket reference guides.	Not reported
Strong [34] (2008)	Nurse - psychoeducation & PST, patient monitoring, communication, liaison between patient, psychiatrist &GP	Antidepressant Prescribing	No role defined	Supervise treatment; Review non-responders; prescribing advisory role. Weekly nurse supervision.	Nurse contacted GP for medication initiation / change and advice from the psychiatrist	Nurse: written materials, tutorials and supervised practice over 3 months.	All nurse sessions video recorded; 10% assessed for adherence
Ell [37] (2008)	Social workers (CDCS) carry out the majority of treatment: PST; communication with the oncology; translators during psychiatric evaluations; patient navigation/case management.. Note: patients choose first line therapy	No role	Monitoring antidepressants in consultation with psychiatrist in maintenance phase	Supervise treatment, prescribe antidepressants. Weekly CDCS supervision	CDCS interacts via written notes or verbally with the treating oncologist; CDCS and psychiatrist manage patients via a clinical data tracking secure website and weekly telephone supervision sessions	Structured training in PST and the study algorithms	Quality assurance by an independent ‘expert’ on 5 audiotaped SW sessions.
Fann [38] (2009)	DCM (nurse or Clin psych) conduct psychosocial history, provide education and behavioral activation; identify treatment preferences: antidepressants and a structured six- to eight-session PST	Make treatment choices	No role	Encouraged to see patients who presented diagnostic challenges/persistent depression for in-person consultations in the primary care setting.	DCM met weekly with a supervising psychiatrist & primary care physician (PCP) to monitor clinical progress/adjust treatment plans	Not reported	Not reported
Kroenke [39] (2010)	DPCM (nurse) recommends treatment in accordance with evidence-based guidelines; monitors response/adherence.	Nil	detects bothersome symptoms; implements treatment recommendation	Supervises DPCM; advises on complex/nonresponding cases	DPCM met weekly to review cases with the pain-psychiatrist to discuss management issues Contact with oncology not specified	Not reported (though notes that the nurse was trained)	Not reported
Sharpe [40] (2014)	Nurse: psychoeducation &PST, behavioural activation; patient monitoring, communication, liaison patient, psychiatrist &GP	Antidepressant Prescribing	No specific role	Supervise treatment; Review non-responders; prescribing advisory role. Weekly nurse supervision	DCPC states: Regular reports are sent to the GP (with copies to other relevant professionals) which detail the patient’s current antidepressant medication, depression score and progress in treatment. The reports are checked by a supervising psychiatrist before being sent and any recommendations are added regarding changes to antidepressant medication	2-3 month training. Achievement of competency in specific clinical areas (basic oncology, basic psychiatry, advanced communication skills, depression assessment and treatment, suicide risk assessment, problem solving therapy, use of the DCPC treatment manual). Training comprised: tutorials, directed reading, role plays and simulated patient treatment sessions. Assessments were both written and practical	Treatment sessions video-recorded. Supervising psychiatrist watched the video-recordings of each nurse’s early sessions; detailed feedback. Standardised rating sheets for each treatment session type completed by nurses and by the supervisors to determine adherence to the treatment approach. Specified behaviours and proscribed behaviours assessed. An independent researcher rated 10% of DCPC sessions.
Walker [33] (2014)	Nurse coordinates depression care by liaising with all relevant health professionals; symptom monitoring; Provide psychoeducation & PST	Antidepressant Prescribing		Weekly review; Supervise treatment response; prescribing advisory role	Regular reports are sent to the GP (with copies to other relevant professionals) which detail the patient’s current antidepressant medication, depression score and progress in treatment. The reports are checked by a supervising psychiatrist before being sent and any recommendations are added	Achievement of competency, includes tutorials, directed reading, roleplay activities, stimulation patient sessions.	Random sample of 10% video recordings of treatment sessions, rated for adherence to treatment manual + quality of delivery
Steel [34] (2016)	Provision of CBT, telephone and face to face,recommendations for pharmacological management dependent on patient preferences, communication of patient preferences/change symptoms to medical team/primary physician	Primary care could manage antidepressant prescribing	Oncologist may manage antidepressant prescribing	Weekly supervision between clinical psychologists and care coordinators - to assess adherence to protocol.	The care coordinators provided information to the medical team about any changes in a patient’s symptoms that might have warranted changes in treatment, changing medication, or adding psychotherapy. However, the medical team may or may not have accepted the care coordinators’ recommendation	300-page intervention manual- included evaluation of depression and cognitive behavioural symptoms.	Not reported

#### A structured management plan tailored to depression symptom severity

Consistent with the collaborative care model, all interventions reported implementation of a structured management plan. The treatment phase ranged from 6 to 16 weeks and included weekly [33, 34, 36–40] or bi-weekly [35] review meetings between the care co-ordinator and psychiatrist to monitor patient progress and make treatment adjustments as required. Seven studies incorporated a stepped care protocol [36, 40] and/or documented criteria for dose escalation and/or psychiatry consultation. [31, 33–35, 37] Of the five studies reporting follow-up post the initial treatment phase, management varied from 3 to 4 months [33, 36] to 8–12 months [37, 38, 40] with non-responders being offered additional psychological treatment booster sessions. [33, 36, 37, 40] Table 2 outlines intervention management plans.

#### Scheduled patient follow-ups

While the majority of interventions included weekly or fortnightly face to face follow up during the treatment phase, one study relied on 3 telephone calls over 12 weeks to supplement automated symptom monitoring [39] and a second study included 2 weekly telephone calls and monthly face to face follow up. [34] The maintenance phase ranged from 3 to 12 months with monthly follow up, [31, 34–37, 40] although one study linked follow up to regular (unspecified) oncology clinic visits. [37] The web-based symptom monitoring study did not include any maintenance or relapse prevention strategies after the initial treatment period, although symptoms continued to be monitored online. [34] Follow up protocols are listed in Table 2.

#### Enhanced inter-professional communication

Across all studies communication was facilitated by the care co-ordinator and there were clear communication protocols between the care co-ordinator and the supervisory psychiatrist/psychologist regarding treatment review and follow up. However, there was limited inter-professional collaboration to establish the treatment plan and discuss progress incorporated into any of the collaborative care intervention models. For the three SMART studies, where the GP was responsible for prescribing anti-depressants, the care co-ordinator sent detailed written reports outlining depression scores, progress and any treatment recommendations to each GP. Although these studies did not detail any formal communication with oncologists, copies of reports were forwarded to *other relevant professionals*. [33, 36, 40]. Similarly, for studies where the oncologist was responsible for anti-depressant prescribing, the protocols included feedback from the care co-ordinator to the oncologist, [35, 39] although the nature of this feedback was only documented in one study. [37] For two studies the inter-professional communication plan was not specified. [34, 38] Across studies, engagement with the psychiatrist was typically limited to their review of the reports, with recommendations subsequently communicated by the care co-ordinator, although in one study the psychiatrist was available to provide same day treatment decision support on an ad-hoc basis. [35] Case conferencing with the non-mental health care providers was not a feature in any of the collaborative care models described. Communication protocols are listed in Table 3.

#### Quality of study reporting

Risk of bias was assessed based on Cochrane criteria. (Additional file 3: Table S3). Randomisation processes were well described in six studies. [33, 35–38, 40]. Across studies patients and the health professionals delivering care were not blinded to group allocation, however researchers assessing outcomes remained blinded. Sample size considerations were reported for four studies [33, 36, 39, 40] and met by three studies. [36, 39, 40] Rates of attrition at the end of the initial treatment phase (3–4 months) were reported for three studies [33, 35, 36] and ranged from 2 to 20%. Attrition during the maintenance phase (6–9 months) was reported for five studies [33, 34, 37–39] and ranged from 4 to 57%. Long term attrition rates ≥12 months was reported for 4 studies (9–45%) [37–40]. Analysis was based on intention to treat for seven studies. [33–36, 38–40]

## Discussion

Collaborative care interventions have the potential to improve management of depression for people with cancer. However, before wider implementation, there are several important questions that need to be addressed, including clearer definition of the elements of collaborative care (including roles, communication processes, governance), which collaborative care components are essential to treatment outcomes and who is best placed to deliver care. We sought to explore these questions by systematically describing the components of depression collaborative care interventions. Eight collaborative care RCTs were identified in this review. Five interventions were developed specifically for patients with cancer and three interventions were adapted from a more general collaborative care intervention for management of depression in older patients.

All studies identified in our review highlighted the benefit of systematic, intensive depression treatment and ongoing symptom monitoring, and reported decreased depression symptoms in comparison to usual care. However, in contrast to collaborative care interventions more generally, the review found the interventions were primarily delivered in the hospital setting rather than in primary care. While the hospital-based care model makes sense for those receiving ongoing cancer treatment, this model does little to address the preference of patients for care closer to their home and issues of access, particularly for those patients not in active treatment. [43] Similarly, the continued dependence on hospital specialists for the management of patients does not ameliorate the scarcity of psycho-oncology resources available in many cancer services.

In the literature, collaborative care is used to describe a range of interventions of varying intensity from simple interventions to encourage compliance with medication (typically delivered via telephone) to complex multi-component interventions that incorporate psychological and pharmacological treatments and intensive monitoring and follow up. [29] The studies identified in this review were consistent with this heterogenous depiction of collaborative care, ranging from symptom monitoring and patient navigation, anti-depressant adherence models to interventions involving proactive follow-up by care co-ordinators working closely with psychiatrists integrating pharmacological medical and psychological treatments as part of individualized treatment plans. As a result, treatment dose within the interventions identified varied in level of intensity as well as whether patients were able to access psychological and pharmacological treatment. Importantly, a number of collaborative care models were reliant on anti-depressant medication as the primary treatment. This is of concern given current gold standard depression treatment recognises the importance of combined cognitive behavioural therapy and anti-depressant medication, [44] and the fact that patients may be reluctant to take antidepressant medication. [45] Despite this, all studies documented clear pathways to patient management and follow up protocols to ensure patients had access to appropriate levels of care.

Despite the focus of hospital-based care, the single study in our review that provided care in the primary care setting, [38] demonstrated the acceptability among people diagnosed with cancer to attend primary care appointments for depression management. Other studies such as the Sharpe study [40] purported to provide the option for patients to choose depression care in either the cancer centre or primary care, however in truth this was limited to anti-depressant prescribing rather than overall psychological treatment and care coordination, and the authors did not evaluate differences in outcome based on whether GPs or oncologists were responsible for anti-depressant management. Lack of GP engagement across studies is surprising given the increasing evidence GPs can play an important role in collaborative care models in terms of continuity of care and reduced patient burden due to excessive travel. From a health service perspective, inclusion of GPs will in part address some of the psycho-oncology workforce shortages that mean that many patients do not receive depression treatment in a timely manner. Under this model of care, less complex patients’ pharmacological treatment can be managed in the community, with psychiatry treating only those patients with persistent symptoms.

A key feature of collaborative care models is a team-based approach to delivery of care. Previous reviews have highlighted the importance of a multi-disciplinary approach to care, with inclusion of case managers with mental health training reported to increase the efficacy of the model. [29] Our review found in the context of cancer, care co-ordination was primarily undertaken by nurses trained to deliver the intervention. However, given their limited specialist mental health training and consistent with the findings of previous work conducted by our Group that nurses are inadequately equipped to deliver depression care, [46] there was a high level of psychiatry case management incorporated into all studies, with psychiatrists providing both staff supervision and treatment decision-making. The time commitment required for ongoing psychiatry oversight limits long term sustainability of the proposed model in routine care as it is predicated on sufficient psychiatry staff to provide the high level of supervision. Modifications of the model to include other (non-medical) mental health staff such as clinical psychologists, such as demonstrated in the study by Steel [34] in the delivery of psychological treatment and supervision of care co-ordinators in a stepped care model, with psychiatric review reserved for those patients in need of more intensive psychiatric care, is one way to reduce is one way to reduce the need for routine psychiatry oversight.

Additionally, over-reliance on specialist psychiatrist involvement for treatment decision-making further underscores the perception among oncology clinicians that the mental wellbeing of patients is not the responsibility of the cancer team and is further reinforced when oncologists are informed of their patient’s depression diagnosis but not engaged in any delivery of depression care. Similarly, although primary care physicians provided pharmacological treatment to their patients as part of the intervention, we were unable to identify any studies that incorporated specific training for GPs about prescribing and follow up. Upskilling of non-mental health providers in evidence-based management of depression is essential for a truly collaborative model to be sustainable. However, in the studies identified in this review, communication pathways between hospital-based care providers and GPs failed to engage GPs in shared decision-making as much of the communication was via written reports or telephone calls initiated by the care co-ordinator (nurse). This resulted in passive engagement with treatment decision-making whereby GPs implemented recommendations for individual patients rather than establishing ongoing communication where treatment decisions were jointly shared between care providers, taking into account not only the patient’s cancer and current depression episode, but each patient’s wider medical and social history available from their primary care provider. Attention to clinical governance and management of shared care is necessary to achieving positive patient outcomes. The funding models and organisation of primary care and specialist care vary internationally and influence the governance of such models. This context necessitates closer examination of the processes and ingredients of findings regarding collaborative care when evaluating transferability to diverse settings. Evidence from “shared care” for primary mental disorders indicates that substantial cross-organisational commitment is required, with consistently delivered interventions, and with close attention to staff selection, training and supervision. [47] Implementation and longterm sustainability of shared care models is contingent on funding models that support clinical pathways that incorporate primary and tertiary care, the introduction of information technology to facilitate information sharing, and collaborative interdisciplinary practice models.

## Study limitations

The results of this review need to be considered in light of a number of limitations. Firstly, we were unable to conduct the planned meta-analysis to compare between intervention components due to the small number of studies identified. The review however does extend the recent meta-analysis conducted by Li and colleagues [30] that demonstrated overall efficacy of the model, as we were able to qualitatively explore the components of each intervention and compare the roles of health professionals across interventions. Our analysis of intervention components was however limited by the intervention descriptions detailed in study publications; the authors may have used a more detailed intervention protocol when conducting their study and explicit references to previously published standardised protocols were included in assessment of interventions. Similarly, we were unable to determine the quality of training provided as part of the interventions or assess the quality of care provided by each member of the care team. Generalisability of review results are also limited to interventions published in English. All studies identified also excluded patients with co-morbid psychiatric and substance use disorders, limiting generalisability of the study results given the high prevalence of co-occurring mental health conditions in the general population. Despite these limitations, this review highlights that, collaborative care interventions for depression in oncology do vary according to key criteria such as level of inter-professional engagement and communication, inclusion of psychological and pharmacological treatment options and documented follow up.

## Conclusions

In the context of research, collaborative care interventions for the management of depression in cancer have demonstrated efficacy over usual care. However, the sustainability of the level of hospital staff engagement raises questions about the model’s utility in routine care. Current models of care reflect hospital-based multi-disciplinary models of care. Greater engagement with oncology and primary care is required to reduce the over-reliance on specialist psychiatry services. Models that utilise existing community-based services, including clinical psychologists in partnership with GPs have the potential to enhance depression care. Given the aim of collaborative models is to provide more care in the community, future models need to consider the training needs of primary care providers and there needs to be greater emphasis placed on inter-professional communication and shared decision-making.

Future research is required to explore patient and provider acceptability as well as pragmatic non-inferiority trial designs that incorporate process evaluations to identify implementation strategies and practice change required to move these models of care from research into clinical practice.

## Additional files

Additional file 1: **Table S1.** example search strategy PsycINFO search. (DOCX 13 kb)

Additional file 2: **Table S2.** Reasons for study exclusion from the review at both title and abstract and fulltext screening. (DOCX 17 kb)

Additional file 3: **Table S3.** Assessment of Bias and Outcomes. (DOCX 16 kb)

## Abbreviations

ADAPT-C: Alleviating Depression Among Patients with Cancer intervention
CBT: Cognitive behavioural therapy
DCPC: Depression Care for People with Cancer intervention
GPs: General practitioners/primary care physicians
IMPACT: Improving Mood – Promoting Access to Collaborative Treatment intervention
MDD: Major depressive disorder
PST: Problem solving Treatment
