# Supplementary material for: Is care really shared? A systematic review of collaborative care (shared care) interventions for adult cancer patients with depression
Source: BMC Health Serv Res. 2019 Feb 14;19:120. doi: 10.1186/s12913-019-3946-z (PMC6376792; doi:10.1186/s12913-019-3946-z)
Supplement: Supplementary file 1 — Table S1. example search strategy PsycINFO search. (DOCX 13 kb) [file 12913_2019_3946_MOESM1_ESM.docx]

Database: PsycINFO Search Strategy:

--------------------------------------------------------------------------------

1 dysthm*.tw.

2 (subclinical adj2 depressi*).tw.

3 (subsyndromal adj2 depressi*).tw.

4 (subthreshold adj2 depressi*).tw.

5 (subdiagnostic adj2 depressi*).tw.

6 Depression/

7 depressive disorder/ or depressive disorder, major/ or dysthymic disorder/

8 depressive disorder.mp. [mp=title, abstract, heading word, table of contents, key concepts, original title, tests & measures]

9 exp depressive disorder/ or exp dysthymic disorder/

10 major depression.mp.

11 dysthymic disorder.mp. or exp dysthymic disorder/

12 MDD.mp. [mp=title, abstract, heading word, table of contents, key concepts, original title, tests & measures]

13 persistent depressive disorder.mp. [mp=title, abstract, heading word, table of contents, key concepts, original title, tests & measures]

14 (unipolar adj3 depress*).mp. [mp=title, abstract, heading word, table of contents, key concepts, original title, tests & measures]

15 exp Affective Disorders/

16 or/1-15

17 cancer.mp.

18 (tumour: or tumor: or neoplas:).mp

19 exp neoplasms/

20 carcinoid.mp.

21 carcinoma*.mp. [mp=title, abstract, heading word, table of contents, key concepts, original title, tests & measures]

22 metasta*.mp. [mp=title, abstract, heading word, table of contents, key concepts, original title, tests & measures]

23 adenocarcinoma*.mp. [mp=title, abstract, heading word, table of contents, key concepts, original title, tests & measures]

24 sarcoma*.mp. [mp=title, abstract, heading word, table of contents, key concepts, original title, tests & measures]

25 or/17-24

26 (collaborat* adj2 care).mp. [mp=title, abstract, heading word, table of contents, key concepts, original title, tests & measures]

27 (share* adj2 care).mp. [mp=title, abstract, heading word, table of contents, key concepts, original title, tests & measures]

28 (integrat* adj2 care).mp. [mp=title, abstract, heading word, table of contents, key concepts, original title, tests & measures]

29 (joint adj2 care).mp. [mp=title, abstract, heading word, table of contents, key concepts, original title, tests & measures]

30 or/26-29

31 16 and 25 and 30

32 clinical trials/ or "treatment outcome clinical trial".md. or ((randomi?ed adj7 trial*) or ((single or doubl* or tripl* or treb*) and (blind* or mask*)) or (controlled adj3 trial*) or (clinical adj2 trial*)).ti,ab,id.

33 (((comprehensive* or integrative or systematic*) adj3 (bibliographic* or review* or literature)) or (meta-analy* or metaanaly* or "research synthesis" or ((information or data) adj3 synthesis) or (data adj2 extract*))).ti,ab,id. or ((review adj5 (rationale or evidence)).ti,ab,id. and "Literature Review".md.) or (cinahl or (cochrane adj3 trial*) or embase or medline or psyclit or pubmed or scopus or "sociological abstracts" or "web of science").ab. or ("systematic review" or "meta analysis").md.

34 32 or 33

35 31 and 34

36 limit 35 to (English language)
