# Supplementary material for: Is care really shared? A systematic review of collaborative care (shared care) interventions for adult cancer patients with depression
Source: BMC Health Serv Res. 2019 Feb 14;19:120. doi: 10.1186/s12913-019-3946-z (PMC6376792; doi:10.1186/s12913-019-3946-z)
Supplement: Supplementary file 2 — Table S2. Reasons for study exclusion from the review at both title and abstract and fulltext screening. (DOCX 17 kb) [file 12913_2019_3946_MOESM2_ESM.docx]

Supplementary Table 2 Reasons for Exclusion

| **Exclusion on Abstract and Title** | |
| --- | --- |
| **Author/Date** | **Reason for exclusion** |
| Aljohani (2015) | Not collaborative care  Condition not depression |
| Archer etal (2012) | Condition not cancer |
| Ben-Arye etal (2011) | Not collaborative care  Condition not depression |
| Bichard etal (2016) | Condition not cancer |
| Cheville etal. (2018) | Condition not depression |
| Dauchy etal (2012) | Non-randomised  Not collaborative care |
| Deng etal (2013) | Not collaborative care |
| El-Jawahri etal (2017) | Focus on carers |
| Evans & Connis (1995) | Not collaborative care |
| Fujisawa etal (2015) | Not collaborative care  Condition not depression |
| Hoodin etal. (2013) | Condition not cancer  Not collaborative care |
| Hyle etal (2014) | Condition not cancer |
| Jeitler etal. (2017) | Non-randomised |
| Johansson etal. (2001) | Not collaborative care  Not depression |
| Kaasa etal. (2008) | Non-randomised  Condition not depression |
| King (2017) | Letter/Editorial  Non-randomised |
| Kissane etal. (2016) | Not collaborative care  Non-randomised |
| Kulcher et al (2007) | Not collaborative care  Condition not depression |
| Li etal. (2016) | Non-randomised |
| Li etal. (2017) | Reviews/Metanalysis |
| Luckett etal (2011) | Not collaborative care |
| McLachlan etal. (2001) | Not collaborative care  Not depression |
| McGorry (2005) | Condition not cancer |
| McPheeters etal. (2012) | Condition not cancer |
| Moorey et al (2009) | Not collaborative care |
| Nezu et al (2003) | Condition not depression |
| Nipp etal. (2016) | Not collaborative care  Condition not depression |
| Northouse etal. (2012) | Focus on carers |
|  |  |
| Nottelmann etal. (2017) | Condition not depression |
| O'Malley etal. (2014) | Condition not cancer |
| Pottie etal. (2012) | Condition not cancer  Not collaborative care |
| Quick & Kiefer. (2013) | Not collaborative care |
| Radbruch etal. (2011) | Not collaborative care |
| Rhondali etal. (2010) | Not collaborative care  Non-randomised |
| Ross (2005) | Not collaborative care  Not depression |
| Savard et al (2006) | Not collaborative care |
| Schulman-Green etal. (2016) | Not collaborative care |
| Shannahoff-Khalsa (2005) | Not collaborative care |
| Simms etal. (2011) | Not collaborative care |
| Sylvia etal. (2015) | Condition not depression |
| Syrjala etal. (2014) | Condition not depression |
| Temel etal. (2017) | Non-randomised  Not collaborative care |
| Temel etal. (2010) | Condition not depression  Not collaborative care |
| Towler etal. (2013) | Condition not depression |
| Walker etal. (2014) | Non-randomised |
| Walker etal. (2014) | Non-randomised |
| Yano etal. (2012) | Not collaborative care  Condition not cancer |
| **Exclusion after Full Text Review** | |
| Artherholt & Fann. (2012) | Reviews/Metanalysis |
| Breland etal. (2015) | Reviews/Metanalysis |
| Caruso etal. (2014) | Not collaborative care |
| Clarke & Currie (2009) | Not collaborative care |
| Copsey etal. (2015) | Secondary data |
| Craven & Bland (2006) | Condition not cancer |
| Duarte etal. (2015) | Secondary data |
| Ell etal. (2010) | Secondary data |
| Ell etal. (2010) | Secondary data |
| Ell etal. (2007) | Non-randomised |
| Emery etal. (2014) | Condition not cancer |
| Geerse etal. (1990) | Condition not depression |
| Hall etal. (2014) | Non-randomised |
| Hart etal. (2012) | Reviews/Metanalysis |
| Klafke etal. (2015) | Condition not depression |
| McCorkle etal. (2015) | Not collaborative care |
| Meijer etal. (2013) | Reviews/Metanalysis |
| Mitchell & Harvey (2014) | Not collaborative care |
| Narasimhan etal. (2008) | Not collaborative care |
| O'Connor etal. (2010) | Secondary data |
| Panagioti etal. (2016) | Reviews/Metanalysis Secondary data |
| Shimizu (2013) | Reviews/Metanalysis |
| Smith etal (2007) | Not collaborative care |
| Smith etal. (2008) | Condition not depression |
| Steel etal. (2011) | Non-randomised |
| Steel etal. (2013) | Condition not cancer |
| Steel etal. (2014) | Reviews/Metanalysis |
| Steel etal. (2016 | Condition not depression |
| Steel etal. (2014) | Secondary data |
| Walker & Sharpe (2009) | Non-randomised |
| Watson etal. (2013) | Reviews/Metanalysis |
