# Supplementary material for: Is care really shared? A systematic review of collaborative care (shared care) interventions for adult cancer patients with depression
Source: BMC Health Serv Res. 2019 Feb 14;19:120. doi: 10.1186/s12913-019-3946-z (PMC6376792; doi:10.1186/s12913-019-3946-z)
Supplement: Supplementary file 3 — Table S3. Assessment of Bias and Outcomes. (DOCX 16 kb) [file 12913_2019_3946_MOESM3_ESM.docx]

Supplementary Table 3: Assessment of Bias and Outcomes

| **Reference** | **Adequate sequence generation** | **Adequate allocation concealment** | **Blinding addressed (participants, personnel, assessors)** | **Sample Size considerations** | **Incomplete outcome data addressed (Attrition)** | **Screening Criteria** | **Primary Outcome Measure** | **Analysis** | **Results** |
| --- | --- | --- | --- | --- | --- | --- | --- | --- | --- |
| Dwight-Johnson et al (2005) | Computer-generated random assignment | Yes | Assessors | Not listed | At 4 or 8 month follow up : 36 completed assessment (65%) | MDD, dysthymia or persistent depressive symptoms; PHQ-9 scores in Mild | PHQ-9 | ITT | OR: 4.51 (95% CI = 1.07-18.93; p=.03) |
| Strong et al (2008) | Minimisation for sex, age, primary cancer site and extent of disease | No | Assessors | 200; 80% power, 5% significance to find CSD of 0·21 and 5% loss to follow-up | At 3 months: 196 remained (98%) | HADS >15, SCID via telephone | SCL-20 | ITT | OR: 0.34 (95% CI = 0.13-0.55) |
| Ell et al (2008; 2011) | Computer-generated random assignment | Yes | Assessors | Not listed | At 6 months: 318 patients remained (67%)  At 12 months: 258 patients remained (55%)  At 24 months: 210 patients remained (44%) | Adults, patients with more than 90 days after cancer diagnosis | PHQ-9 | Not listed | OR: 2.09 (95% CI = 1.13-3.86; p=.02) |
| Fann et al (2009) | Computer-generated random assignment | Yes | Assessors | Not listed | At 6 months: 207 remained (96%)  At 12 months: 195 remained (91%)  At 18 months: 189 remained (88%)  At 24 months: 183 remained (85%) | Depression as identified by SCID DSM IV | SCL-20 | ITT | OR=2.69 (95% CI = 1.54-4.71) |
| Kroenke et al (2010) | Computer-generated random assignment | No | Assessors | 97 per symptom group; 80% power to detect a 20% absolute difference in response rates with a 2-tailed α <.05. 80% power to detect a moderate ES of 0.4 when analyzing depression and pain as continuous outcomes | At 1 month: 354 remained (87%)  At 3 months: (82%)  At 6 months: (75%)  AT 12 months: (66%) | PHQ-9 (>10),  Endorsement of either depressed mood, anhedonia or both. | HCL-20 | ITT | ES: 0.41 |
| Sharpe et al (2014) | Database software algorithm | Yes | Statistician and outcome assessors | two treatment groups of 250; 90% power; 5% signif to detect a difference of at least 0·15 (15%) in the proportion of participants achieving the primary outcome of treatment response | At 48 weeks: 447 remained (89%) | 12 month survival, SCID DSMIV to identify MDD | SCL-20 | ITT | OR: 8.5 (95% CI =5.5 - 13.4; p<.0001) |
| Walker et al (2014) | Database software algorithm | Yes | Statistician and outcome assessors | 150; 90% power , 5% significance to detect SMD 0·53 and 80% power to detect SMD 0·46. The target sample size was revised from 200 | At 3 months: 113 remained (80%)  At 6 months: 97 remained (68%)  At 9 months: 93 remained (65%) | 3 month survival, SCID DSMIV to identify MDD | SCL-20 | ITT | OR: 5.88 (95% CI 2.42-14.33; p<.0001) |
| Steel et al (2016) | Block randomisation via sex and vascular invasion | Yes | Statistician and initial assessor | Not listed | At baseline: 224 remained (86%)  At 6 months: 178 remained (67%) | A psychiatric intake by the care coordinator | CES-D | ITT | ES: 0.71 (at 6 months) |
